# Descriptions of four new species of *Bactrocera* and new country records highlight the high biodiversity of fruit flies in Vietnam (Diptera, Tephritidae, Dacinae)

**DOI:** 10.3897/zookeys.797.29138

**Published:** 2018-11-19

**Authors:** Luc Leblanc, Camiel Doorenweerd, Michael San Jose, Hong Thai Pham, Daniel Rubinoff

**Affiliations:** 1 University of Idaho, Department of Entomology, Plant Pathology and Nematology, 875 Perimeter Drive, MS2329, Moscow, Idaho, 83844-2329, USA; 2 University of Hawaii, Department of Plant and Environmental Protection Services, 3050 Maile Way, Honolulu, Hawaii, 96822-2231, USA; 3 Vietnam National Museum of Nature, Vietnam Academy of Science and Technology, Hoang Quoc Viet St, Hanoi, Vietnam

**Keywords:** Dacini, *
Dacus
*, pest, taxonomy, *
Zeugodacus
*

## Abstract

Recent snap-shot surveys for fruit flies in Vietnam in 2015 and 2017 using traps baited with the male Dacinae fruit fly lures methyl eugenol, cue-lure and zingerone, collected 56 species, including 11 new country records and another 11 undescribed species, four of which are described in this paper. This increases the number of described species known to occur in Vietnam from 78 to 93. Species accumulation curves, based on the Chao 2 mean estimate, suggest that we collected 60–85 % of the local fauna at the sites sampled, and that species diversity decreases with increasing latitude. The four new species are named: Bactrocera (Tetradacus) ernesti Leblanc & Doorenweerd **sp. n.**, B. (Asiadacus) connecta Leblanc & Doorenweerd **sp. n.**, B. (Parazeugodacus) clarifemur Leblanc & Doorenweerd **sp. n.**, and B. (Bactrocera) adamantea Leblanc & Doorenweerd **sp. n.** In addition to morphological data COI DNA sequence data of both the COI-5P and COI-3P mitochondrial DNA gene regions is provided. Three of the four newly described species are morphologically and genetically easily distinguished from all other members of Dacini. *Bactroceraclarifemur***sp. n.** is superficially similar to *B.pendleburyi* (Perkins) based on morphology, but there are several apomorphic characters to distinguish the two. Both COI and a segment of the nuclear gene Elongation Factor 1 alpha separate the two species as well.

## Introduction

Dacini fruit flies are a species rich group distributed throughout the Old World tropics, including remote Pacific islands. It includes 932 described species, of which 83 are pests of fruit and fleshy vegetables, and new species are continuously being discovered (Vargas et al. 2015, Doorenweerd et al. 2018). Extensive surveys for Dacini fruit flies were carried out in Asia in recent decades (Linder and McLeod 2008). Vietnam was particularly well surveyed, with 78 species, including 19 described as new, reported to occur in the country (Drew and Romig 2013, 2016, Table 1).

**Table 1.** Checklist of Dacine fruit flies of Vietnam, including previously known species, new country occurrence records, and number of specimens collected in the surveys (2015–2017) reported in this paper. A complete list of species of Vietnam and its neighboring countries is available on Suppl. material 1: Table S1 (supplementary online material).

Taxon	Lure	Ba Bể	Mê Linh – Tam Đảo	Bạch Mã	Cát Tiên
**Genus *Bactrocera***					
*B.abbreviata* (Hardy)*	ZN	7	110	1579	74
*B.adamantea* Leblanc & Doorenweerd*	ZN	0	0	0	4
*B.aethriobasis* (Hardy)	ME	0	0	0	0
*B.bhutaniae* Drew & Romig	CL	1	15	5	8
*B.bimaculata* Drew & Hancock	CL	0	0	0	0
*B.binhduongiae* Drew & Romig	ME	0	0	0	0
*B.bivittata* Lin & Wang*	ME	0	0	3	2
*B.carambolae* Drew & Hancock ^1^	ME, ZN	0	0	23	68
*B.cibodasae* Drew & Hancock	CL	0	0	0	0
*B.clarifemur* Leblanc & Doorenweerd*	ZN	0	0	4	45
*B.connecta* Leblanc & Doorenweerd*	ZN	0	0	1	3
*B.correcta* (Bezzi) ^1^	ME	0	0	70	4
*B.dongnaiae* Drew & Romig	CL	0	0	0	9
*B.dorsalis* (Hendel) ^1^	ME	1136	1369	1603	946
*B.ernesti* Leblanc & Doorenweerd*	ZN	0	0	6	0
*B.eurycosta* Drew & Romig	CL	0	0	0	0
*B.flavoscutellata* Lin & Wang	CL	0	0	5	0
*B.fulvifemur* Drew & Hancock	CL	0	0	0	0
*B.fuscitibia* Drew & Hancock	CL	0	0	0	0
*B.gombokensis* Drew & Hancock	CL	4	0	0	3
*B.holtmanni* (Hardy)	CL	0	0	0	0
*B.illusioscutellaris* Drew & Romig	CL, ZN	0	1	2	3
*B.jaceobancroftii* Drew & Romig	ME	0	0	0	0
*B.kanchanaburi* Drew & Hancock	ME	0	0	0	193
*B.laithieuiae* Drew & Romig	CL	0	0	0	0
*B.lateritaenia* Drew & Hancock	CL	0	0	11	5
*B.latifrons* (Hendel) ^2^	latilure	0	0	0	0
*B.limbifera* (Bezzi)	CL	0	0	19	0
*B.lombokensis* Drew & Hancock	CL	0	0	0	0
*B.neocognata* Drew & Hancock	CL	0	0	0	0
*B.nigrita* (Hardy)	ME	0	0	0	0
*B.nigrotibialis* (Perkins)	CL	0	0	29	22
*B.osbeckiae* Drew & Hancock	CL	0	0	0	0
*B.paraarecae* Drew & Romig*	ME	0	0	1	9
*B.pendleburyi* (Perkins)*	ZN	0	0	17	1
*B.propinqua* Hardy & Adachi	CL	0	0	7	42
*B.pruniae* Drew & Romig ^3^	No lure	0	0	0	0
*B.pyrifoliae* Drew & Hancock ^2^	No lure	0	0	0	0
*B.quasiunfulata* Drew & Romig	CL	0	0	0	0
*B.raiensis* Drew & Hancock	ME	0	0	0	0
*B.ritsemai* (Weyenbergh)	CL	0	0	0	2
*B.rubigina* (Wang & Zhao)	CL, ZN	16	34	7	3
*B.sapaensis* Drew & Romig	CL	0	0	0	0
*B.syzygii* White & Tsuruta*	ZN	1	1	110	400
*B.thailandica* Drew & Hancock	CL	19	84	2	0
*B.tuberculata* (Bezzi) ^2^	ME	0	0	0	0
*B.umbrosa* (Fabricius)* ^3^	ME	0	0	11	0
*B.usitata* Drew & Hancock	CL	0	0	2	13
*B.verbascifoliae* Drew & Hancock	ME	0	0	0	0
*B.wuzhishana* Li & Wang	ME	0	2	1	0
*B.zonata* (Saunders) ^1^	ME	0	0	0	0
*B.* species 59 (*dorsalis* complex)**	CL	0	0	1	0
*B.* species 74**	ZN	0	0	0	1
*B.* species 104 (near *rubigina*) (ms6131)**	CL	0	0	0	3
*B.* species 105 (near *citima*) (ms6135)**	CL	0	0	0	3
*B.* species 106 (*dorsalis* complex)**	CL	0	0	9	17
**Genus *Dacus***					
*D.bannatus* Wang	CL	0	0	0	0
*D.discretus* Drew & Romig	CL	0	0	0	0
*D.dorjii* Drew & Romig	CL	0	0	0	0
*D.longicornis* (Wiedemann) ^4^	CL	4	0	0	2
*D.satanas* (Hering)	ZN	12	3	0	2
*D.siamensis* Drew & Hancock	CL	0	0	0	0
*D.sphaeroidalis* (Bezzi)	CL	0	0	0	0
*D.tenebrosus* Drew & Hancock*	CL, ZN	0	0	0	2
*D.vijaysegerani* Drew & Hancock	CL, ZN	0	0	0	41
**Genus *Zeugodacus***					
*Z.ablepharus* (Bezzi)	CL	4	0	0	0
*Z.aithonota* (Drew & Romig)	CL	0	0	0	0
*Z.apicalis* (de Meijere)	CL	0	0	1	253
*Z.assamensis* White	CL	0	0	0	0
*Z.atrifacies* (Perkins)	CL	0	0	2	0
*Z.baoshanensis* (Zhang, Ji, Yang & Chen)	CL	0	0	0	0
*Z.caudatus* (Fabricius) ^5^	CL	0	0	0	3
*Z.cilifer* (Hendel)	CL	2	1	0	0
*Z.cucurbitae* (Coquillett) ^4^	CL, ZN	1	0	1	66
*Z.daclaciae* (Drew & Romig)	CL	0	0	0	0
*Z.diaphorus* (Hendel)	CL	0	0	0	1
*Z.diversus* (Coquillett) ^5^	ME	0	0	0	0
*Z.heinrichi* (Hering)	CL, ZN	223	0	66	19
*Z.hoabinhiae* (Drew & Romig)	CL	0	0	0	0
*Z.hochii* (Zia) ^4^	CL, ZN	0	0	0	60
*Z.incisus* (Walker)	CL	2	0	0	5
*Z.infestus* (Enderlein)	CL	143	0	0	0
*Z.isolatus* (Hardy)	CL	0	0	0	0
*Z.khaoyaiae* (Drew & Romig)*	CL	10	0	0	0
*Z.maculifacies* (Hardy)	CL	0	0	0	0
*Z.melanofacies* (Drew & Romig)*	CL	0	0	0	1
*Z.mukiae* (Drew & Romig)	CL	0	0	0	0
*Z.nakhonnayokiae* (Drew & Romig)	CL	0	0	0	0
*Z.ochrosterna* (Drew & Romig)	CL	0	0	0	0
*Z.proprescutellatus* (Zhang, Che & Gao)*	CL	0	0	2	0
*Z.scutellaris* (Bezzi) ^5^	CL	0	0	0	0
*Z.scutellatus* (Hendel) ^5^	CL	41	13	0	0
*Z.sinensis* (Yu, Bai & Chen)*	CL	0	0	1	0
*Z.sonlaiae* (Drew & Romig)	CL	0	0	0	0
*Z.tau* (Walker)	CL	200	36	5	73
*Z.trilineatus* (Hardy)	CL	0	0	0	0
*Z.vultus* (Hardy)	CL	0	0	0	0
*Z.yoshimotoi* (Hardy)	CL	0	0	0	0
*Z.* species 72 (near *infestus*)**	CL	0	0	0	1
*Z.* species 101 (near *tau*)**	CL	0	0	0	2

* New country occurrence records. ** Undescribed new species. ^1^ Polyphagous fruit pest. ^2^ Oligophagous fruit pest. ^3^ Monophagous fruit pest. ^4^ Cucurbit fruit pest. ^5^ Cucurbit flower pest. Pest categorizations after Vargas et al. (2015).

Several advances have been made in recent years towards reconstructing the Dacini tree of life based on molecular data, the results of which are often in conflict with interpretations of the morphology of the flies (Krosch et al. 2012, Virgilio et al. 2015, Dupuis et al. 2017, San Jose et al. 2018). At present, there are no reliable apomorphy-based morphological circumscriptions for three of the four genera in the tribe: *Dacus*, *Bactrocera*, *Zeugodacus* (San Jose et al. 2018). Only *Monacrostichus*, which includes just two species, is recognized from its wing venation and is presumed to represent a basal branch, but no representatives have been included in any molecular phylogenetic studies. The genus *Dacus* is often presented as distinct by having ‘merged’ abdominal tergites (Drew and Romig 2013, 2016) but these are not merged in any physical sense, they just tightly join, hence this is not an absolute character and there are species which display this to a varying extent. The most recent phylogenies based on either a fairly good species coverage (San Jose et al. 2018) or a large amount of genetic data (Dupuis et al. 2017) support a sister relationship between *Dacus* and *Zeugodacus*, with *Bactrocera* as sister to both. Adding more species to these phylogenies will enable further studies of the morphological characters that may be used to recognize members of these genera.

Identifying Dacini flies is of interest not only to taxonomists and systematists, but also to customs officers, pest prevention program employees and farmers. Because of the large size of the group and the high levels of homoplasy between characters and little morphological diversity, morphology-based identifications are currently mostly reserved to specialists. Until we reach a better understanding of the relationships among species that will allow for an assessment of the polarity of morphological characters (San Jose et al. 2018), molecular diagnostics may be a preferred method for identifying potential pest species. Sequencing of the DNA barcoding marker COI (5’P region) has been applied to Dacini for both species identification and determining potential source of invading populations (e.g., Barr et al. 2014, Choudhary et al. 2017, Meeyen et al. 2014). However, the reliability of such approaches heavily depends on the availability of validated reference data (Mutanen et al. 2016). As part of a project to survey the genetic diversity of potential pest species across Asia and further understand phylogenetic relationships globally (Leblanc et al. 2015b, 2016), our team carried out Dacini fruit fly surveys in Vietnam in 2015 and 2017. A number of new country records and undescribed new species were uncovered, greatly aided by the inclusion of the male lure zingerone (Royer 2015, Royer et al. 2017) in the trapping. Four new species, which were also included under temporary species names in a recently published phylogeny (San Jose et al. 2018), are described here.

## Materials and methods

### Collecting and curation

**Figure 1.**
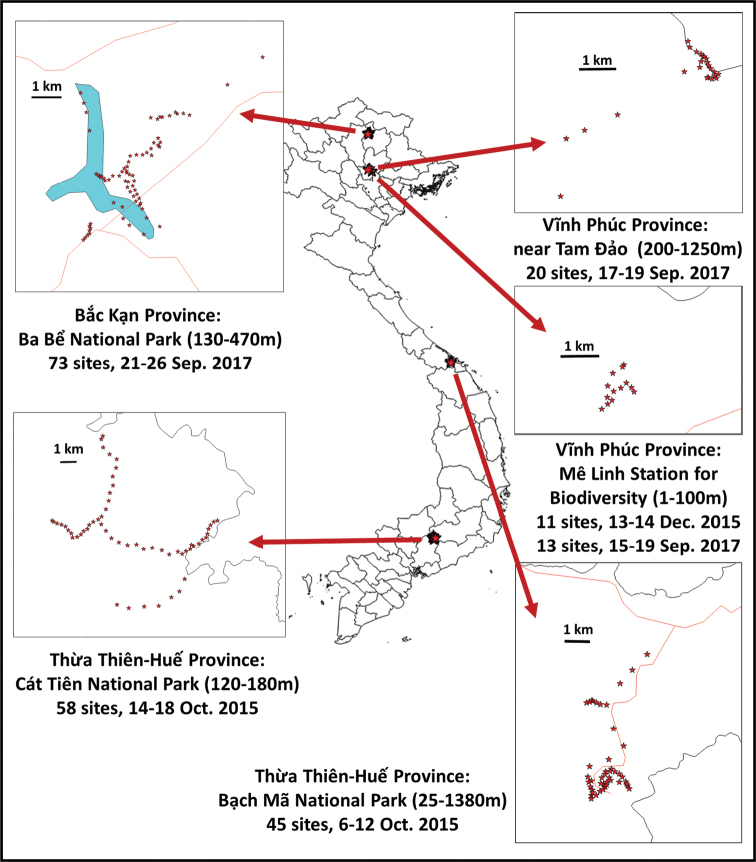
Trapping locations in the Vietnam surveys (2015, 2017).

To collect fruit flies, we used sets of three traps made of modified urine sample cups (described in Leblanc et al. 2015a), separately baited with the fruit fly lures methyl eugenol, cue-lure and zingerone, with a 10×10mm piece of dichlorvos insecticide strip to kill trapped flies. These traps were maintained for three to five days at each of 220 sites in forest reserves and national parks in 2015 and 2017 (Figure 1). Specimens retrieved from the traps were preserved in 95 % ethanol and stored in a -20 °C freezer when returned to the laboratory. A selection of specimens was dried and pinned. We pinned these specimens fresh out of the ethanol each with a minuten pin through the scutum, then soaked them in ethyl-ether for 3–12 hours to preserve the coloration, and double-mounted them. We photographed specimens using a Nikon D7100 camera attached to an Olympus SZX10 microscope. Pictures from different focal plains were merged using Helicon Focus pro v6.7.1. Initially, we performed identifications based on morphology, using available keys (Drew and Hancock 1994, Drew and Romig 2016). Measurements were taken using an ocular grid mounted on an Olympus SZ30 dissecting microscope. The collecting and taxonomy information for all specimens can be found at BOLD http://dx.doi.org/10.5883/DS-VIETDACI.

### Morphological terms

For the morphological terms we generally follow White (1999). We have attempted to avoid comparative terms to describe the size of structures, although this was sometimes necessary to stay consistent with the common practice in Dacini. In particular, two structures that have typically been used to designate subgenera group or genus assignments: the male surstylus posterior lobe size and the concavity of sternum V (Drew and Romig 2013, figure 22; Virgilio et al. 2015, De Meyer et al. 2015). The posterior lobe of the male surstylus is considered ‘long’ when it is twice as long as the anterior lobe, and the concavity is considered ‘deep’ when it reaches midway of the tergum. The application of subgenera in their current sense is debatable, as they are mostly not monophyletic (San Jose et al. 2018). However, to allow for easier comparison with existing literature and in the absence of a better alternative, we assign the newly described species to a subgenus that best fits morphologically. For each species we indicate in the notes section how it may be incorporated into the widely used keys for Dacini in Asia (Drew and Romig 2016).

### DNA extraction, PCR and sequencing

The new species we describe here have been included in a previously published seven-gene molecular phylogeny under temporary species names (San Jose et al. 2018), where the methods for DNA extraction, PCR primers and conditions and sequencing methods were extensively described. For all holotypes we extracted DNA from one dissected hind leg and sequenced the seven genes that were used for the molecular phylogeny: the 3’P side of COI, two fragments of CAD, Wingless, White-eye, PGD, EF1-alpha, and Period. For this study we also sequenced the 5’P side of COI, the DNA barcoding fragment, using the primer pair L1-DCHIM (5’-TCGCCTAAACTTCAGCCATT-3’) and HCO-2198 (5’-TAAACTTCAGGGTGACCAAAAAATCA-3’). Concatenating the 5’P barcoding side and 3’P side of COI resulted in a 1,535 base-pair (bp) long alignment of fragments. Some additional EF1-alpha sequence data were produced for specimens of *B.clarifemur*, using the primers and sequencing conditions as described in San Jose et al. (2018). All sequences are made available both through BOLD (dx.doi.org/10.5883/DS-VIETDACI) and GenBank (accession numbers cited in the BOLD link and accession numbers MG683030–MG684292, previously published in San Jose et al. (2018), with *B.connecta* referred to as *Bactrocera* sp. 68, *B.adamantea* as sp. 69, *B.clarifemur* as sp. 70 and *B.ernesti* as sp. 71). Maximum likelihood trees were generated using FastTree v2.1.5 (Price et al. 2010), implemented in Geneious R10.2.3, using the General-Times-Reversable (GTR) model of nucleotide evolution. To account for the varying rates of evolution across sites, we used 25 rate categories for each site. Support values were calculated with the Shimodaira-Hasegawa test (Shimodaira and Hasegawa 1999) and are indicated on each respective branch. Sample numbers for the taxa refer to the individual voucher numbers of the flies. For the statistical parsimony (TCS) haplotype network based on COI sequence data for the species pair *B.pendleburyi* and *B.clarifemur*, we trimmed the ends of the alignments with ambiguous bases, and reconstructed a network using PopArt (Clement et al. 2002, Leigh and Bryant 2015). The phylogenies presented on Figures 3, 5, 11 are single gene trees and are interpreted as molecular diagnostic tools, for phylogenetic relationships we refer to the published multi-gene phylogenies (San Jose et al. 2018, Dupuis et al. 2017).

### Estimating total diversity

To determine the fraction of the diversity we covered by our sampling, whilst acknowledging the male lure collecting method bias, we used EstimateS (Colwell 2013) to generate species accumulation curves. The estimates are based on the number of species collected, in particular singletons and doubletons, to extrapolate to the diversity with increasing number of samples using the incidence-based Chao 2 algorithm. The Chao 2 indicator was selected because it does not include abundance in its extrapolation, compensating for the abundance bias in our data related to how strongly each species is attracted to the lures that were used, plus, with several species being agricultural pests, the overall diversity abundance likely does not follow a normal distribution. Diversity estimations were done for the overall dataset and for the four broad sampled locations (Figure 1) separately, with 100 randomizations without replacement for confidence intervals.

### Abbreviations

**UHIM** University of Hawaii Insect Museum

**CTAHR**University of Hawaii College of Tropical Agriculture and Human Resources

**USDA** United States Department of Agriculture

**WFBM** University of Idaho’s William F. Barr Entomological Museum

**VNMN** Vietnam National Museum of Nature

## Results

We collected a total of 9,516 specimens, representing 56 species (Table 1). Eleven previously described species, most of which are known to occur in neighboring countries, are new occurrence records for Vietnam. Another 11 species that were collected are either new to science, four of which are described below, or we were otherwise unable to link them to any described species. Our survey results increase the total number of described species in Vietnam from 78 to 93, 10 % of the globally known diversity of Dacini (Doorenweerd et al. 2018). Potentially, an additional 52 species, known to be present in China, Laos, Cambodia, and Thailand, excluding southern Thailand, may be found in Vietnam (Drew and Romig 2013, Leblanc et al. 2016; listed in Suppl. material 1: Table S1).

### Zingerone attraction

Noteworthy is the capture of *B.syzygii*, a species not attracted to the traditional male lures methyl eugenol and cue lure, and previously assumed to be endemic to Sri Lanka. We collected 512 specimens in zingerone-baited traps in all four parks, indicating that it is commonly present. It was also recently collected in Bangladesh (LL, unpublished) and in India (David et al. 2017). A larger distribution for *B.syzygii* could have been expected, considering the common occurrence of *Syzygium* host plants in the region. Zingerone is also a new lure record for multiple species previously not known to be attracted to male lures: *B.abbreviata* (all 1,770 specimens collected in zingerone), *B.illusioscutellaris* (3/6, i.e. 3 of 6 specimens collected in zingerone), *B.pendleburyi* (all specimens), *B.rubigina* (1/60), *D.satanas* (16/17), *D.tenebrosus* (2/2 specimens), *D.vijaysegerani* (all 41 specimens), *Z.heinrichi* (211/308), and *Z.hochii* (15/60). The four new species described in this paper were also drawn to zingerone. Several other species were collected in very small numbers in zingerone traps, and we therefore find it too uncertain to record them as being attracted to zingerone: *B.bhutaniae* (3 specimens), *B.flavoscutellata* (1), *B.gombokensis* (2), *B.kanchanaburi* (1), *B.lateritaenia* (1), *Z.atrifacies* (1), *Z.infestus* (2), *Z.khaoyaiae* (1), and *Z.tau* (4). Even with only a single specimen out of 60 drawn to zingerone, the attraction of *B.rubigina* to that lure was confirmed in surveys carried out in Bangladesh in 2017, with 2,237 specimens collected in cue-lure and 63 collected in zingerone-baited traps (LL, unpublished).

### Diversity estimates

**Figure 2.**
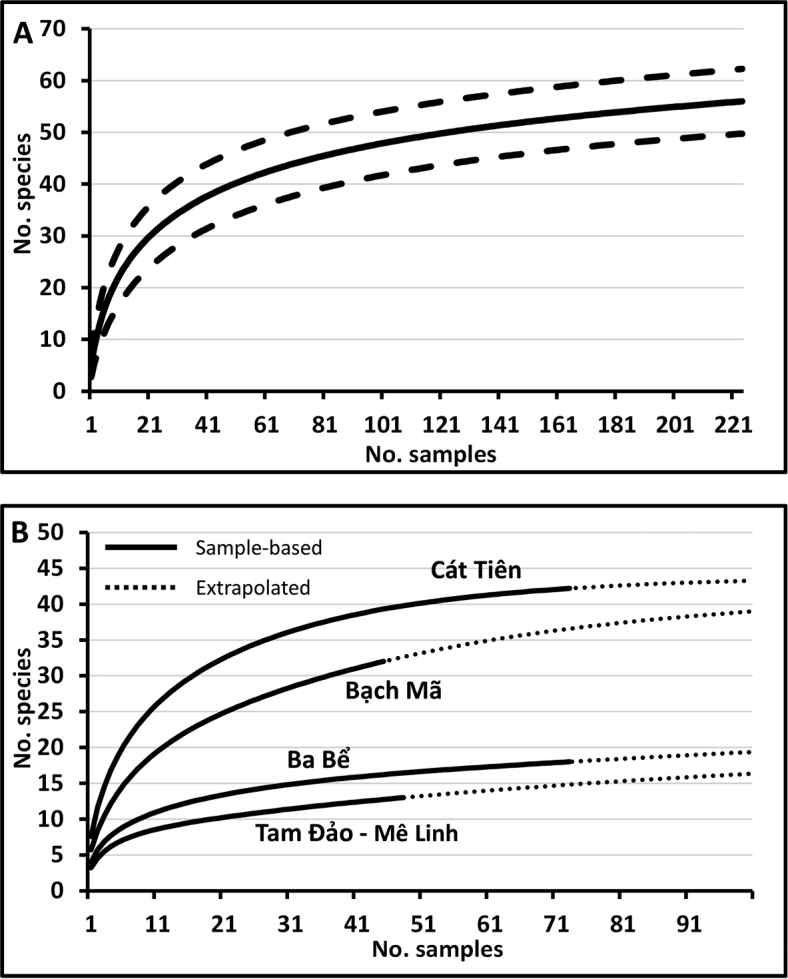
Species accumulation curves for species collected in fruit fly surveys (2015, 2017) across the entire region (**A**), and for the four broad surveyed locations (**B**).

A species accumulation curve, generated using all data across the four locations (Figure 2A), suggest that the 56 species collected represent 60–85 % of the local fauna attracted to the three male lures methyl eugenol, cue lure and zingerone, with a Chao 2 mean estimate of 65.0 species (95 % CI = 58.1–93.9). By far the highest species diversity was collected and projected in central and southern Vietnam: Cát Tiên with 41 species and a mean estimate of 43.1 (41.3–54.2), and Bạch Mã with 32 species and a mean estimate of 39.3 (33.7–63.1) (Figure 2B). The northern sites yielded a lower diversity, despite intense sampling effort (117 trapping sites): Ba Bể with 18 species and a mean estimate of 21.0 (18.4–40.7) and Tam Đảo and Mê Linh together with 13 species, with mean estimate of 16.0 (13.4–35.6). These estimates are based on species attracted to male lures. It is likely that a broader diversity will be uncovered with the regular use of zingerone in trapping and through host fruit surveying. The four new species described below were all collected in zingerone-baited traps.

#### Bactrocera (Tetradacus) ernesti

Taxon classificationAnimaliaDipteraTephritidae

Leblanc & Doorenweerd sp. n.

http://zoobank.org/DC201837-F7AE-487B-86C0-D09B903F4CA7

##### Holotype.

Male. Labelled: “Vietnam: Thừa Thiên-Huế Province, Bạch Mã National Park, 16.2297N, 107.8494E, 6–8-x-2015, M. San Jose and D. Rubinoff, FF485, zingerone lure. Molecular voucher ms6192”. Deposited in UHIM. **Paratypes**: 5 males. Same data as holotype. All specimens pinned and one specimen is molecular voucher ms6255. Three of the paratypes are deposited at UHIM, one at WFBM and one at VNMN.

##### Differential diagnosis.

*Bactroceraernesti* is similar to other members of the subgenus Tetradacus in having an elongate oval abdomen with a petiolate base [oval in most *Bactrocera*] with separate terga [tightly joined in *Dacus*], and a slight concavity of sternum V and short surstylus lobe in the males. It is most similar to *B.minax* and *B.brachycera*, but differs from both in lacking a lateral yellow band connecting the postpronotal lobes to the notopleural suture, the absence of medial postsutural vitta, the anteriorly convergent lateral postsutural vittae, the lightly infuscate wing, and absence of distinct costal band, and the black bands on every abdominal segment.

##### Molecular diagnostics.

**Figure 3.**
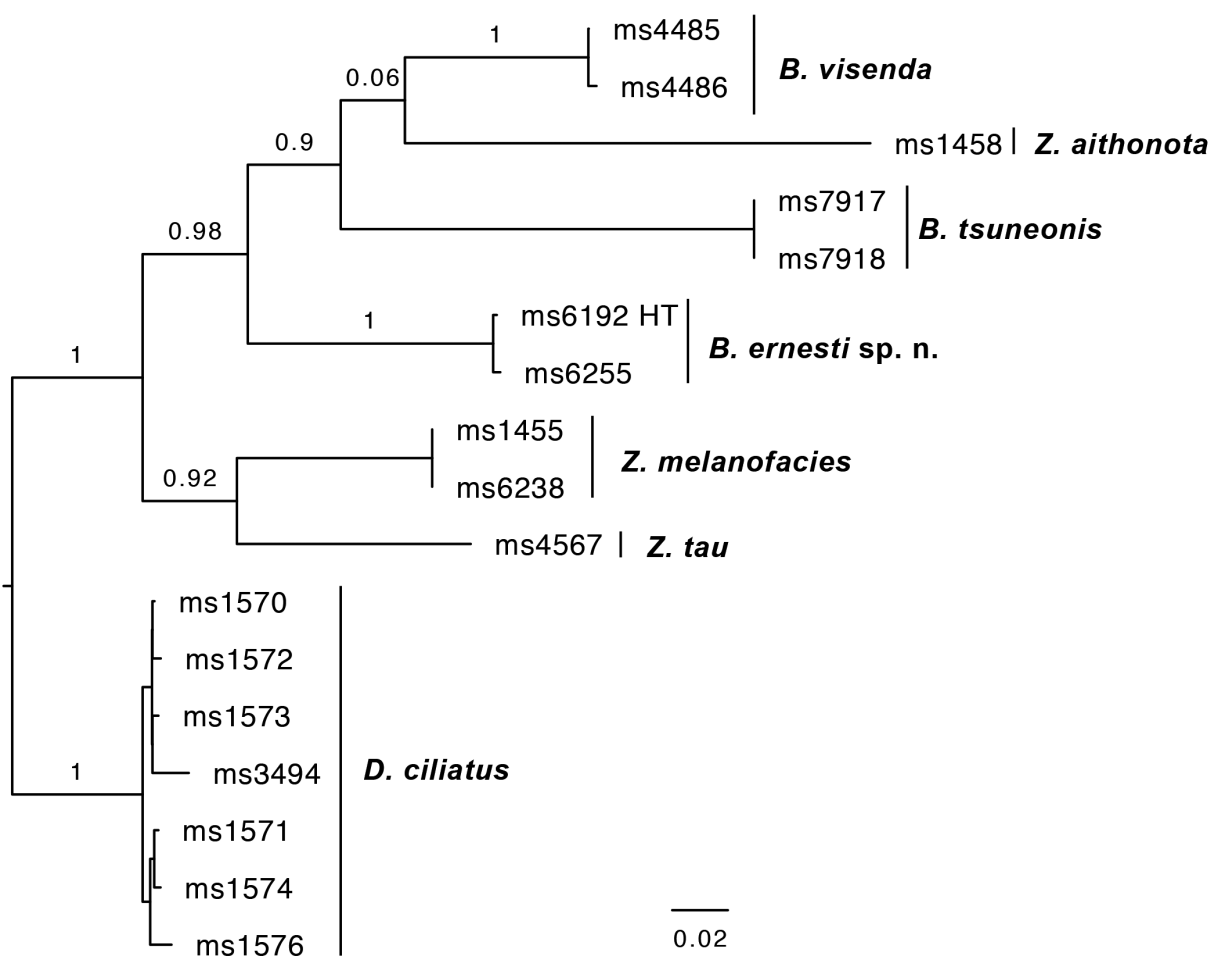
Maximum likelihood tree based on COI sequences of *B.ernesti* sp. n. and several of its genetically closest neighbors, which include *Bactrocera*, *Zeugodacus* and *Dacus* species. Bootstrap branch supports shown for intraspecific relationships. Abbreviations: HT holotype.

*B.ernesti* sp. n. was referred to as *Bactrocera* species 73, represented by the holotype, in the seven-gene phylogeny presented in San Jose et al. (2018). Based on the sampling therein, its closest relative is B. (Tetradacus) tsuneonis (Miyake). The closest relative we could identify based on COI alone is the Australian species *B.visenda*, at a minimum intraspecific pairwise genetic distance of 13.52 % [14.89 % in COI5P and 12.27 % in COI3P] (Figure 3).

##### Description of adult.

**Figure 4.**
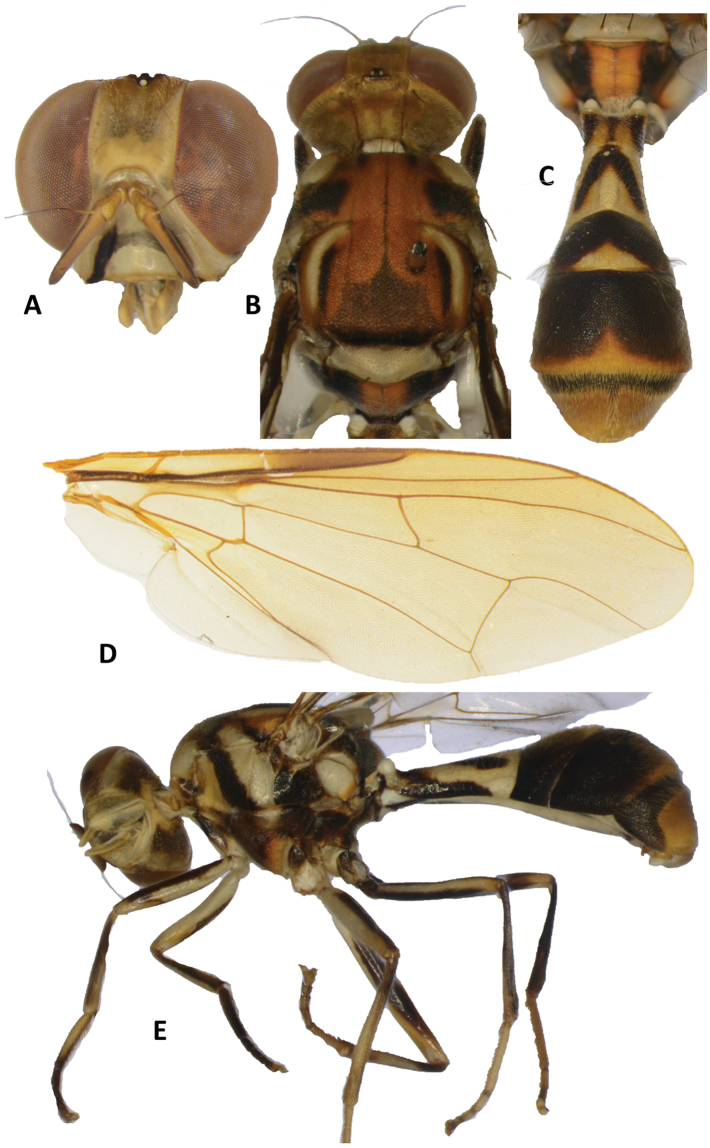
Bactrocera (Tetradacus) ernesti. **A** head **B** head and scutum **C** abdomen **D** wing **E** lateral view.

*Head* (Figure 4A). Vertical length 1.82 ± 0.04 (*SE*) (1.75–1.85) mm. Frons, of even width, length 1.16 ± 0.05 (1.10–1.26) times breadth; uniformly fulvous; anteromedial hump and frons covered by short red–brown hairs; orbital and frontal setae, when present, red–brown: orbital setae absent or at most very weak and frontal setae very weak and may be restricted to superior pair; lunule fuscous. Ocellar triangle black. Vertex fulvous. Face fulvous with elongate black spot in lower half of each antennal furrow, connected by a faint narrow fuscous band across mid height of face; length 0.69 ± 0.02 (0.65–0.70) mm. Genae fulvous, with fuscous sub–ocular spot; one large and numerous smaller red–brown setae present. Occiput fulvous with fuscous markings laterally on its lower half; occipital row with 9–13 dark setae and an irregular inner row of finer setae. Antennae with segments 1 (scape) and 2 (pedicel) fulvous, segment 3 (first flagellomere) fulvous with pale fuscous on outer surface; a strong red–brown dorsal seta on segment 2; arista black (fulvous basally); length of segments: 0.19 ± 0.01 (0.18–0.20) mm; 0.26 ± 0.02 (0.23–0.28) mm; 0.76 ± 0.04 (0.70–0.83) mm.

*Thorax* (Figure 4B, E). Scutum red–brown except a broad light fuscous lanceolate pattern on its posterior third, anteriorly prolonged into three very narrow lines reaching anterior margin, narrow elongate dark fuscous bands adjacent to inner margins of lateral postsutural vittae, broad lateral dark fuscous markings behind postpronotal lobes and two short and narrow parallel dark fuscous bands between postpronotal lobes. Pleural areas dark fuscous except red–brown below postpronotal lobes, anterior half of anepisternum and posterior portion of katepisternum. Yellow markings as follows: postpronotal lobes; notopleura; medium sized and parallel-sided mesopleural (anepisternal) stripe, reaching anterior margin of notopleuron, continuing to katepisternum as a transverse spot, anterior margin straight; entire katatergite except red–brown narrowly along posterior margin; lower quarter to half of anatergite (remainder dark fuscous and red-brown on posterior margin of lower quarter to half); two moderately broad parallel sided lateral postsutural vittae ending shortly before intra-alar setae and curved slightly inwards along notopleural suture. Postnotum medially red–brown and laterally black. Scutellum yellow except for narrow dark fuscous basal band. Setae: 2 scutellar; 1 intra-alar; 1 posterior supra-alar; 1 mesopleural; 4 notopleural; 4 or 6 scapular (often a second pair of median scapular, just behind each bristle); anterior supra-alar and prescutellar bristles absent; all setae well developed and red–brown.

*Legs* (Figure 4E). Fore coxae yellow with outer face dark fuscous; fore trochanters and mid coxae and trochanters yellow; hind coxae and trochanters dark fuscous. Femora yellow with broadly fuscous outer and inner surfaces. Fore and mid tibiae fulvous with dark fuscous on outer face of fore tibia and around base of mid tibia; hind tibiae dark fuscous. Tarsi fulvous with dark fuscous fore tarsomeres 2–5 and ventral face of fore basitarsus. Mid-tibiae each with an apical black spur.

*Wings* (Figure 4D). Length 7.56 ± 0.21 (7.22–7.78) mm; basal costal (bc) cell infuscate and costal (c) cells mostly colorless except at apex; microtrichia along costal margin of cell bc and along costal margin and outer corner of cell c; remainder of wings colorless except fuscous subcostal cell, and lightly infuscate membrane between R_1_ and R_4+5_; supernumerary lobe weakly developed.

*Abdomen* (Fig. 4C, E). Elongate oval and petiolate; terga free; pecten present on tergum III; posterior lobe of surstylus short; abdominal sternum V with a slight concavity on posterior margin. Tergum I as long as wide and tergum II and sterna I and II longer than wide. Tergum I medially fuscous with a faint narrow fuscous medial longitudinal band and laterally black. Tergum II yellow with a large inverted V-shaped medial marking. Tergum III black with large apical triangular yellow marking. Tergum IV black with apical fulvous band with a medial expansion; tergum V fulvous with a narrow basal black band expanded laterally to half the tergum length. Abdominal sterna dark, except yellow sternum II.

##### Etymology.

This species is named after Ernest James Harris (1928–2018), in honor of his long career working as a fruit fly ecologist for the USDA (1962–2006). Some of Dr. Harris’s important contributions include the field implementation of the first eradication program against invasive fruit flies in the Mariana Islands, the establishment of Mediterranean fruit fly suppression programs in North Africa and Chile and studies on its ecology and SIT in Hawaii, as a prelude to the initiation of the ongoing SIT program to prevent its establishment in California, and the development of mass rearing techniques of the fruit fly parasitoid *Fopiusarisanus* (Sonan). He published over 120 scientific papers and was honored with distinctions by the State of Arkansas Black Hall of Fame (1999), the NAACP Hawaii Chapter (2012), the Alpha Phi Alpha Fraternity National award (2013), the US Congressional Gold Medal (2016), the USDA-ARS Hall of Fame (2017), and as CTAHR Outstanding Alumnus (2017). His emergence as African American from a modest cotton farming family in Arkansas to an internationally respected prominent scientist, through hard work and his love for his research, makes Ernie a true role model for the senior author of this paper. Biographic sketches of Dr. Harris were published by Riddick et al. (2015) and Leblanc and Vargas (2018, in press).

##### Notes.

*Bactroceraernesti* keys to couplet 2, page 314, in Drew and Romig (2016), where it can be differentiated from *B.minax* and *B.brachycera* based on the characters mentioned in the differential diagnosis.

#### Bactrocera (Asiadacus) connecta

Taxon classificationAnimaliaDipteraTephritidae

Leblanc & Doorenweerd sp. n.

http://zoobank.org/E534D6C5-300A-4A64-95C7-FE8EAF1AA134

##### Holotype.

Male. Labelled: “Vietnam: Thừa Thiên–Huế Province, Bạch Mã National Park, 16.2098N, 107.8632E, 6-12-x-2015, M. San Jose and D. Rubinoff, FF494, zingerone trap.” ms6195 Deposited in UHIM. **Paratypes**: 3 males. Vietnam, Lâm Đồng Province, Cát Tiên National Park, 16–18-x-2015, at the following sites, identified by their geographical coordinates: 11.4485N, 107.4416E, (1 pinned, molecular voucher ms6105), 11.4447N, 107.4372E, (2 pinned, one molecular voucher ms6239). All specimens collected by Michael San Jose and Dan Rubinoff in zingerone baited traps. One paratype deposited at UHIM, one at WFBM and one at VNMN.

##### Differential diagnosis.

*Bactroceraconnecta* shares morphological features common to all members of *Asiadacus* (absence of prescutellar and anterior supra-alar setae, scutellum with one pair of setae, and males with long posterior lobe on surstylus, slight concavity of sternum V and pecten of cilia present on the abdominal tergum III). It is distinguished from other members of *Asiadacus* by the elongate facial spots along antennal furrows and the small to extensive fuscous transverse band on face (Fig. 6A, B), the broad, apically expanded costal band (Figure 6H, I), and the absence of a medial longitudinal band and presence of extensive lateral black markings on the abdomen (Figure 6D–G).

##### Molecular diagnostics.

**Figure 5.**
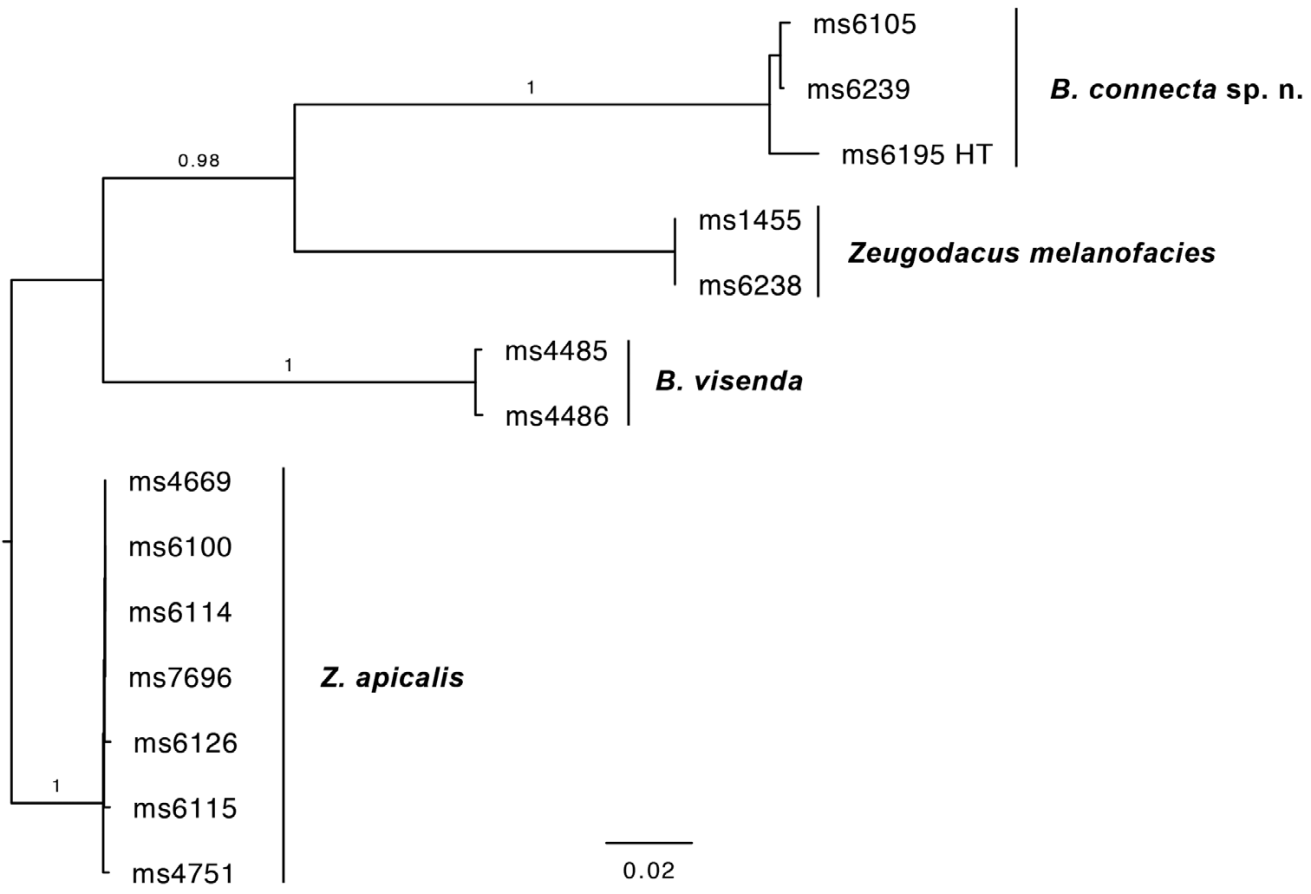
Maximum likelihood tree based on COI sequences of *B.connecta* sp. n. and several of its genetically closest neighbors, which include both *Bactrocera* and *Zeugodacus* species. Bootstrap branch supports shown for intraspecific relationships. HT = holotype.

*Bactroceraconnecta* was referred to as *Bactrocera* species 68, sister to B. (Apodacus) visenda (Hardy), in the seven-gene phylogeny presented in San Jose et al. (2018). The nearest neighbor we could identify based on COI sequence data is *Zeugodacusmelanofacies*, at 15.34 % minimum pairwise intraspecific genetic distance [16.18 % in COI5P and 14.47 % in COI3P] (Figure 5).

##### Description of adult.

**Figure 6.**
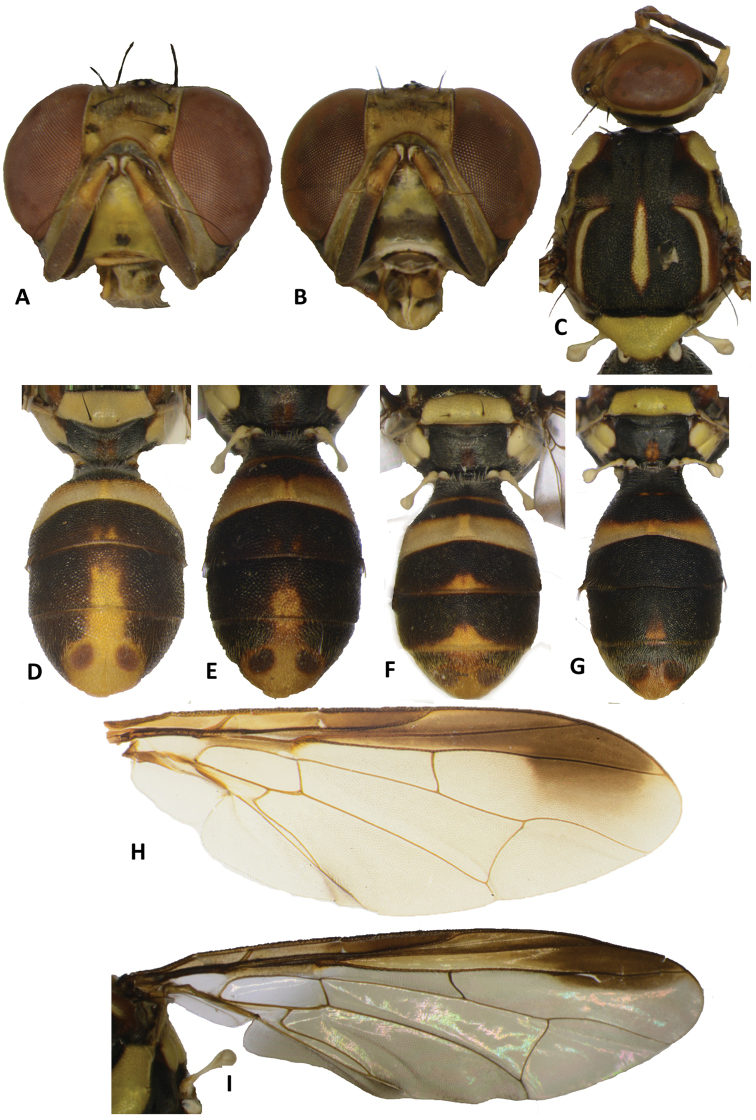
Bactrocera (Asiadacus) connecta. **A, B** head **C** head and scutum **D, E, F, G** abdomen variation **H, I** wing.

*Head* (Figure 6A, B). Vertical length 1.64 ± 0.23 (*SE*) (1.45–1.88) mm. Frons, of even width, length 1.13 ± 0.07 (1.07–1.22) times as long as broad; fulvous with fuscous around orbital setae and on anteromedial hump; latter covered by short red–brown hairs; orbital setae black: one pair of superior and two pairs of inferior fronto-orbital setae present; lunule yellow. Ocellar triangle black. Vertex fuscous. Face fulvous with elongate black spots in each antennal furrow and a fuscous band across lower half of face varying from a small medial spot (Figure 6A) to an entire band across face (Figure 6B); length 0.63 ± 0.09 (0.53–0.75) mm. Genae fulvous, with dark fuscous sub-ocular spot; black seta present. Occiput light to dark fuscous (fulvous laterally in one specimen) and yellow along eye margins; occipital row with 9–11 dark setae. Antennae with segment 1 (scape) fulvous, segments 2 (pedicel) fulvous and fuscous on outer surface, and segment 3 (first flagellomere) fuscous and narrowly fulvous on inner surface; strong red–brown dorsal seta on segment 2; arista black (fulvous basally); length of segments: 0.23 ± 0.02 (0.20–0.25) mm; 0.34 ± 0.02 (0.33–0.38) mm; 0.76 ± 0.07 (0.70–0.83) mm.

**Figure 7.**
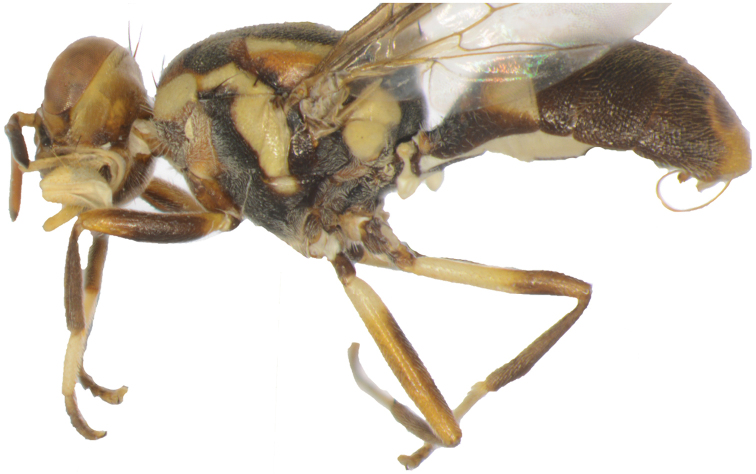
Bactrocera (Asiadacus) connecta: lateral view.

*Thorax* (Figs 6C, 7). Scutum black except sometimes with red–brown as limited markings behind postpronotal lobes and anterior to lateral postsutural vittae and area below postsutural vittae. Pleural areas black except usually red–brown anterior portion of anepisternum. Yellow markings as follows: postpronotal lobes and notopleura (notopleural callus), usually connected by a lateral longitudinal band; medium sized mesopleural (anepisternal) stripe, reaching midway between anterior margin of notopleura and anterior notopleural seta dorsally, continuing to katepisternum as a transverse spot, anterior margin convex; anatergite (posterior apex black); anterior 75 % of katatergite (remainder black); moderately broad medial postsutural vitta beginning at level of or slightly anterior of prescutellar setae and ending at or slightly beyond the level of notopleural suture; two moderately broad parallel sided lateral postsutural vittae ending at or shortly before intra-alar setae and curved inwards along notopleural suture. Postnotum black with apical median red–brown spot. Scutellum yellow except for narrow black basal band. Setae (number of pairs): 1 scutellar; prescutellar absent; 1 intra-alar; 1 posterior supra-alar; anterior supra-alar absent; 1 mesopleural; 2 notopleural; 4 scapular; all setae well developed and red–brown.

*Legs* (Figure 7). Femora fulvous, except for dark fuscous ventral faces of basal three quarters of fore femur, apical three quarters of mid femur and apical two–fifths of hind femur, and yellow basal quarter of mid femur and basal three–fifths of hind femur; tibiae dark fuscous, with or without ventral faces narrowly fulvous; mid-tibiae each with an apical black spur; tarsi fulvous.

*Wings* (Figure 6H, I). Length 5.75 ± 0.67 (5.11–6.67) mm; basal costal (bc) and costal (c) cells fuscous and both covered with microtrichia; remainder of wings colorless except broad fuscous costal band nearly confluent with R_4+5_ and broadly expanded apically; anal streak absent; dense aggregation of microtrichia around A_1_ + CuA_2_; supernumerary lobe of medium development.

*Abdomen* (Figs 6D–G, 7). Elongate oval; terga free; pecten present on tergum III; posterior lobe of surstylus long; abdominal sternum V with a slight concavity on posterior margin. Tergum I and sterna I and II wider than long. Tergum I black. Tergum II yellow with sub-basal or basal broad medial transverse black band that may be extended to cover entire basal half of tergum. Terga III–V black with fulvous areas as small markings on apex of tergum III and narrow median area on tergum IV (or restricted to small apical median marking), continued on base of tergum V and expanded around ceromata. A pair of dark fuscous ceromata (shining spots) on tergum V. Abdominal sterna black, except fulvous sternite II.

##### Etymology.

The species name is an adjective that refers to the longitudinal yellow band connecting the postpronotal lobes and notopleura.

##### Note.

We have tentatively assigned *B.connecta* to *Asiadacus* based on the following combination of characters: absence of prescutellar and anterior supra-alar setae, scutellum with one pair of setae, a long posterior lobe of the male surstylus, slight concavity of sternum V and pecten of cilia present on the abdomen (Drew 1989, Drew and Romig 2016). All known species of *Asiadacus* were transferred to *Zeugodacus*, based on the surstylus and sternum V concavity characters which made them part of the *Zeugodacus* group of subgenera (de Meyer et al. 2015). Only more recently was one species, B. (Asiadacus) apicalis, genetically confirmed to belong to *Zeugodacus* (San Jose et al. 2018). In that same molecular phylogeny, however, *B.connecta* is only distantly related to *B.apicalis* and is placed in the basal grade of subgenera in *Bactrocera*. Recently, Hancock and Drew (2017, 2018) transferred all but two species of *Asiadacus* to the subgenera *Parasinodacus*, *Sinodacus* and *Zeugodacus*, leaving in *Asiadacus* the two species with a large, oval apical wing spot that reaches but does not cross vein M. Because *B.connecta* falls within *Bactrocera* in the recently published phylogeny (San Jose et al. 2018), it cannot be assigned to any of these three subgenera. Moreover, in *B.connecta* the lateral postsutural vittae are not extended as yellow markings beyond the notopleural suture, a character shared by the *Zeugodacus* group of subgenera and genus *Dacus*, referred to as “small notopleural xanthines” by White (2006) and “lateral yellow triangles that reach the notopleural lobes” by Hancock and Drew (2018). Because *B.connecta* cannot be fitted in existing subgenera and to avoid creating a new subgenus, we arbitrarily assign this species to the subgenus Asiadacus sensu Drew and Romig (2016), until the subgeneric relationships within *Bactrocera* are more clearly elucidated. It cannot be keyed beyond couplet 4, page 48, in Drew and Romig (2016), where it can be singled out by the costal band on the wing overlapping R_2+3_ and expanding apically (Figs 6H, I).

#### Bactrocera (Parazeugodacus) clarifemur

Taxon classificationAnimaliaDipteraTephritidae

Leblanc & Doorenweerd sp. n.

http://zoobank.org/22A6F281-868E-4FC5-9959-545E5F8BCBCE

##### Holotype.

Male. Labelled: “Vietnam: Thừa Thiên–Huế Province, Cát Tiên NP, 11.4480N, 107.3826E, 14-18-x-2015, M. San Jose and D. Rubinoff, FF540, zingerone lure, molecular voucher ms6176”. Deposited in UHIM. **Paratypes**: 48 males. Vietnam, Thừa Thiên-Huế Province, Bạch Mã National Park, 6–8-x-2015, at the following sites, identified by their geographical coordinates: 16.2279N, 107.8557E, (1 in ethanol, molecular voucher ms6191), 16.1943N, 107.8490E, (2 in ethanol, including molecular voucher ms6194), 16.2006N, 107.8481E, (1 pinned). Vietnam, Lâm Đồng Province, Cát Tiên National Park, 14–18-x-2015, at the following sites, identified by their geographical coordinates: 11.4920N, 107.3855E, (7 pinned), 11.4867N, 107.3834E, (1 in ethanol, molecular voucher ms6175), 11.4764N, 107.3817E, (2 in ethanol, including molecular voucher ms6249), 11.4715N, 107.3809E, (2 in ethanol), 11.4480N, 107.3826E, (1 in ethanol, molecular voucher ms6176), 11.4436N, 107.3925E, (8 in ethanol), 11.4412N, 107.4026E, (3 pinned), 11.4419N, 107.4080E, (1 in ethanol, molecular voucher ms6177), 11.4394N, 107.4241E, (5 pinned and 2 in ethanol, including molecular vouchers ms6094 and ms6095, in ethanol), 11.4531N, 107.3557E, (1 in ethanol), 11.4486N, 107.3584E, (1 in ethanol), 11.4564N, 107.3686E, (1 in ethanol), 11.4381N, 107.4267E, (1 in ethanol), 11.4398N, 107.4290E, (1 in ethanol), 11.4539N, 107.4430E, (4 pinned), 11.4485N, 107.4416E, (3 pinned); 11.4472N, 107.4392E, (1 pinned). All specimens collected by Michael San Jose and Daniel Rubinoff in zingerone baited traps. All paratypes are deposited at UHIM, except five at WFBM and three at VNMN.

##### Differential diagnosis.

*Bactroceraclarifemur* (Figure 9A–G) is genetically and morphologically closely related to *B.pendleburyi* (Perkins) (Figure 10A–E). Both share the defining characters of subgenus Parazeugodacus (two pairs of scutellar setae; male with lateral pecten on tergum III (though present or absent in different species of *Parazeugodacus* according to Hancock and Drew 2015), a slight concavity on posterior margin of abdominal sternum V, and posterior lobe of male surstylus short), as well as the absence of medial postsutural vitta and the entirely yellow scutellum. *Bactroceraclarifemur* differs from *B.pendleburyi* in that all femora are entirely fulvous (Figure 9G), whereas apices of all femora in *B.pendleburyi* are apically broadly dark (Figure 10E).

##### Molecular diagnostics.

**Figure 8.**
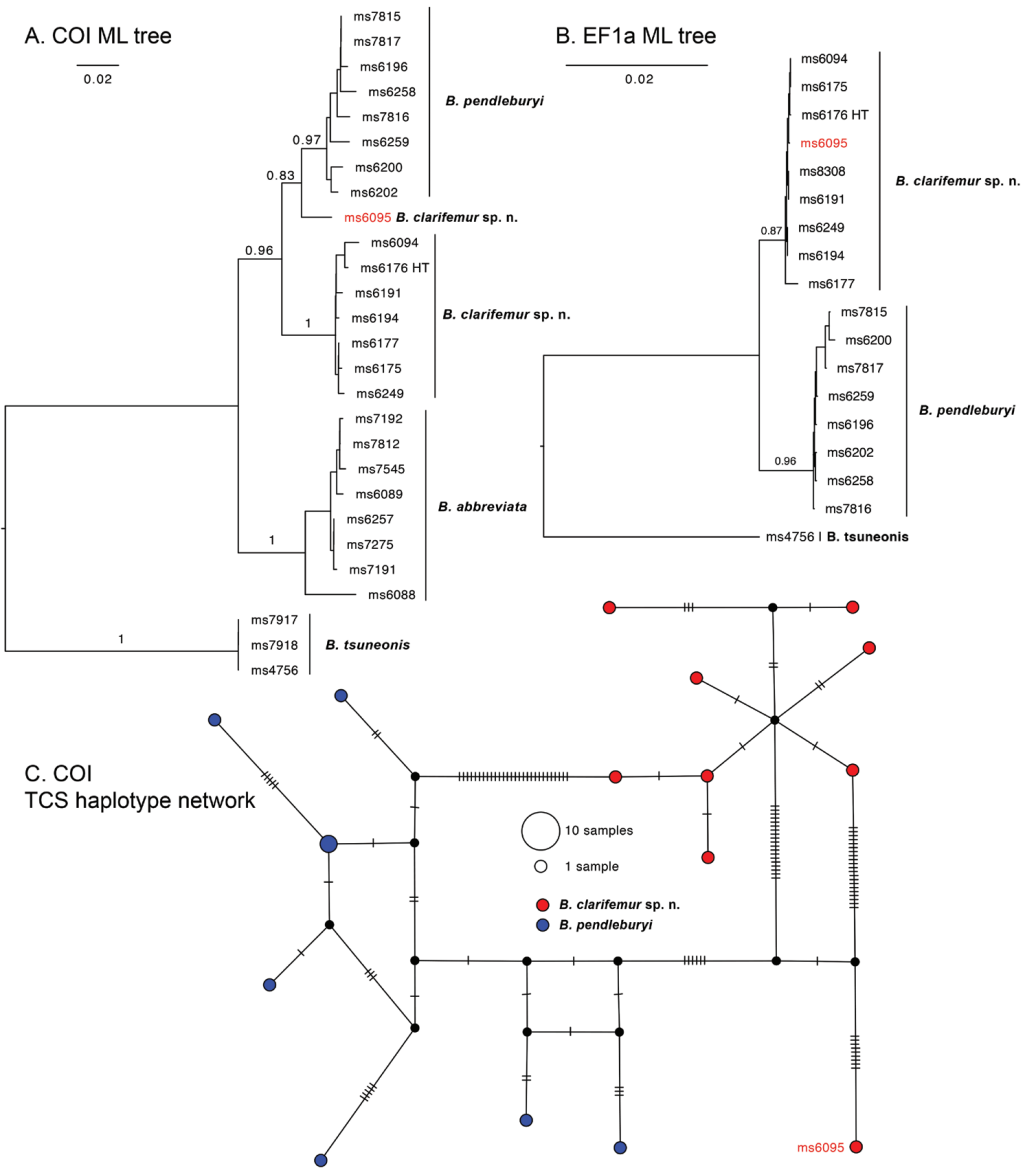
Maximum likelihood trees and haplotype network based on COI (A) and EF1a (B) sequences of *B.clarifemur* sp. n. and several of its genetically closest neighbors. C displays a TCS haplotype network, with notches on the connections to indicate mutations, based on COI data of *B.clarifemur* and *B.pendleburyi* and shows that specimen ms6095 is relatively distantly related from all others. Bootstrap branch supports shown for intraspecific relationships. Abbreviation: HT holotype.

**Figure 9.**
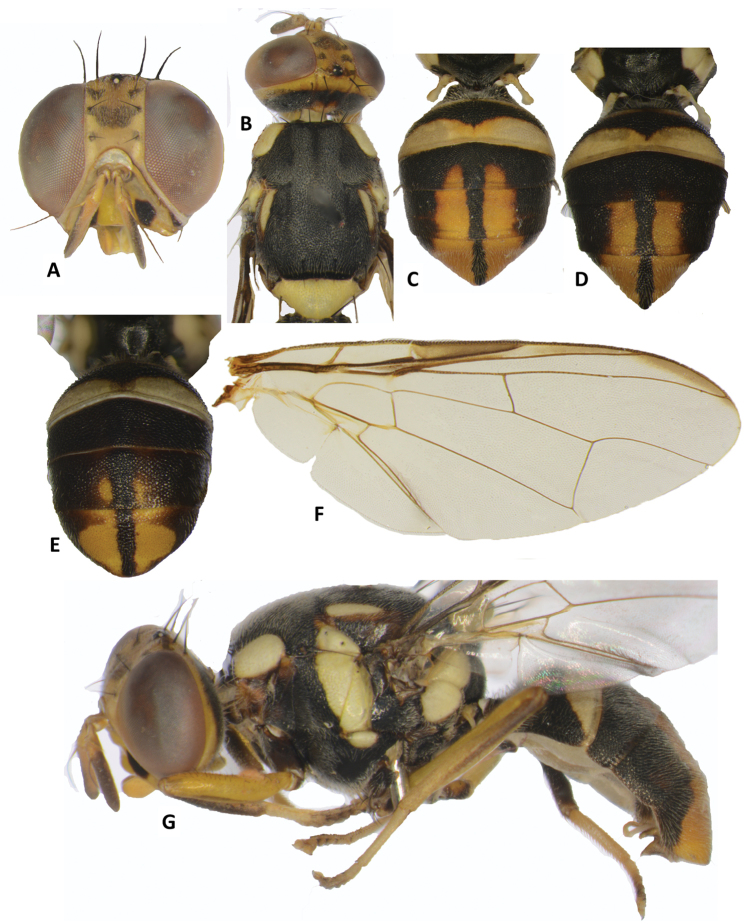
Bactrocera (Hemigymnodacus) clarifemur. **A** head **B** head and scutum **C, D, E** abdomen variation **F** wing **G** lateral view.

This species was referred to as *Bactrocera* sp 70 and is a close relative yet distinctly monophyletic sister to B. (Parazeugodacus) pendleburyi, in the San Jose et al. (2018) seven-gene phylogeny. Based on COI sequence data the two species are in two monophyletic clusters, with one exception. We found an aberrant COI haplotype from specimen (ms6095) that groups with *B.pendleburyi* in ML analysis, though at a minimum of 2.66 % pairwise genetic distance from any of the other specimens (Figure 8). Morphologically, specimen ms6095 fits with *B.clarifemur* (Figure 9A–G). We sequenced an additional nuclear gene to confirm the genetic distinction. Based on EF1a sequences, both species, with specimens from the same localities, were separated in monophyletic groups with a minimum pairwise distance of 1.35 %. A haplotype network of COI sequences further shows that specimen ms6095 is relatively distantly related to both and may eventually be found to represent a cryptic species. The nearest neighbor to both *B.pendleburyi* and *B.clarifemur* is *B.abbreviata* at 6.91 % minimum intraspecific pairwise distance in COI.

**Figure 10.**
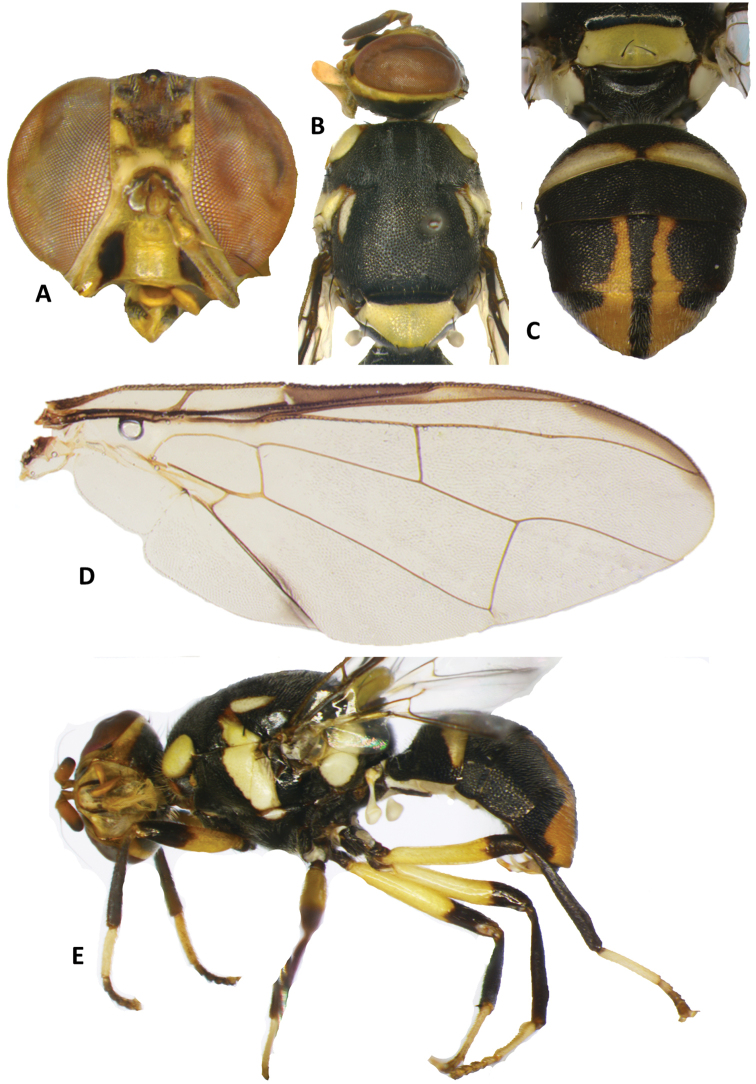
Bactrocera (Hemigymnodacus) pendleburyi. **A** head **B** head and scutum **C** abdomen **D** wing **E** lateral view.

##### Description of adult.

*Head* (Figure 9A). Vertical length 1.40 ± 0.13 (*SE*) (1.20–1.55) mm. Frons, of even width, length 1.32 ± 0.06 (1.22–1.43) times as long as broad; fulvous with usually fuscous spot around orbital setae and on anteromedial hump; latter covered by short red–brown hairs; orbital setae dark fuscous: one pair of superior and two pairs of inferior fronto-orbital setae present; lunule yellow. Ocellar triangle black. Vertex fuscous. Face fulvous with medium sized oval black spots in each antennal furrow; length 0.47 ± 0.04 (0.40–0.53) mm. Genae fulvous, with fuscous sub-ocular spot; red–brown seta present. Occiput dark fuscous and yellow along eye margins; occipital row with 5–7 dark setae. Antennae with segments 1 (scape) and 2 (pedicel) fulvous and segment 3 (first flagellomere) fulvous with pale fuscous on outer surface; a strong red–brown dorsal seta on segment 2; arista black (fulvous basally); length of segments: 0.21 ± 0.02 (0.18–0.23) mm; 0.24 ± 0.01 (0.23–0.25) mm; 0.62 ± 0.05 (0.53–0.68) mm.

*Thorax* (Figure 9B, G). Scutum black and frequently red–brown below lateral postsutural vittae. Pleural areas black and usually narrowly red–brown on anterior corner and frequently posterior corner of proepisternum anterior to postpronotal lobes. Yellow markings as follows: postpronotal lobes; notopleura (notopleural callus); medium sized mesopleural (anepisternal) stripe, reaching anterior margin of notopleura dorsally, continuing to katepisternum as a transverse spot, anterior margin slightly convex; anatergite (posterior apex black); anterior 60 % of katatergite (remainder black); two moderately broad and short postsutural vittae, tapering posteriorly and ending a short distance behind anterior intra-alar setae. Postnotum black. Scutellum yellow except for narrow black basal band. Setae (number of pairs): 2 scutellar; 1 prescutellar; 1 intra-alar; 1 posterior supra-alar; 1 anterior supra-alar; 1 mesopleural; 1 notopleural; 4 scapular; all setae well developed and black.

*Legs* (Figure 9G). Femora entirely fulvous; fore and hind tibiae dark fuscous, mid tibia basally dark fuscous and apically becoming fulvous; mid-tibiae each with an apical black spur; tarsi fulvous.

*Wings* (Figure 9F). Length 4.68 ± 0.35 (4.22–5.00) mm; basal costal (bc) and costal (c) cells colorless; microtrichia in outer corner of cell c only; remainder of wings colorless except fuscous subcostal cell, very narrow light fuscous costal band confluent with R_2+3,_ not widened apically and ending just slightly past the extremity of R_4+5_; anal streak absent; dense aggregation of microtrichia around A_1_ + CuA_2_; supernumerary lobe of medium development.

*Abdomen* (Figure 9C–E). Oval; terga free; pecten present on tergum III; posterior lobe of surstylus short; abdominal sternum V with a slight concavity on posterior margin. Tergum I and sterna I and II wider than long. Tergum I dark fuscous with a narrow transverse fulvous band across posterior margin but not reaching lateral margins; tergum II dark fuscous with a transverse posterior fulvous band which just reaches the narrow black posterolateral corners; tergum III dark fuscous; terga IV–V orange–brown with broad medial dark fuscous band reaching the apex of abdomen and broad lateral dark fuscous bands narrowed in apical half of tergum V. Orange–brown markings and medial band frequently extended to apical portion of tergum III. Ceromata (shining spots) on tergum V orange–brown and indistinct. Abdominal sterna dark, except for fuscous sternite II.

##### Etymology.

The name is an adjective that refers to the absence of dark markings on the femora.

##### Notes.

The characters distinguishing *B.clarifemur* and *B.pendleburyi* were noted as variation of *B.pendleburyi* by Drew and Romig (2013), genetic evidence hereby confirms that they indicate distinct species.

#### Bactrocera (Bactrocera) adamantea

Taxon classificationAnimaliaDipteraTephritidae

Leblanc & Doorenweerd sp. n.

http://zoobank.org/9813AF40-899B-4DFE-A80C-A839B3474860

##### Holotype.

Male. Labelled: “Vietnam: Lâm Đồng Province, Cát Tiên National Park, Ranger station Road, 11.4485N, 107.4416E, 16–18-x-2015, M. San Jose and D. Rubinoff, FF581, Zingerone trap. Molecular voucher ms6092”. Deposited in UHIM. **Paratypes**: 3 males. Vietnam, Lâm Đồng Province, Cát Tiên National Park, 16–18-x-2015, at the following sites, identified by their geographical coordinates: 11.4539N, 107.4430E, (1 pinned, molecular voucher ms6093), 11.4485N, 107.4416E, (1 pinned, molecular voucher ms6236), 11.4472N, 107.4392E, (1 in ethanol). All specimens collected by Michael San Jose and Daniel Rubinoff, in zingerone-baited traps. One paratype deposited in UHIM, one in WFBM and one in VNMN.

##### Differential diagnosis.

*Bactroceraadamantea* belongs to the (polyphagous) *B.dorsalis* complex of notoriously difficult to identify species (Leblanc et al. 2015c), defined by having a mostly dark scutum, a costal band that does not expand apically, and a black T shaped pattern on the abdomen. It can easily be differentiated from all congeners however by the yellow diamond shaped medial vitta on the scutum (Figure 12B).

##### Molecular diagnostics.

**Figure 11.**
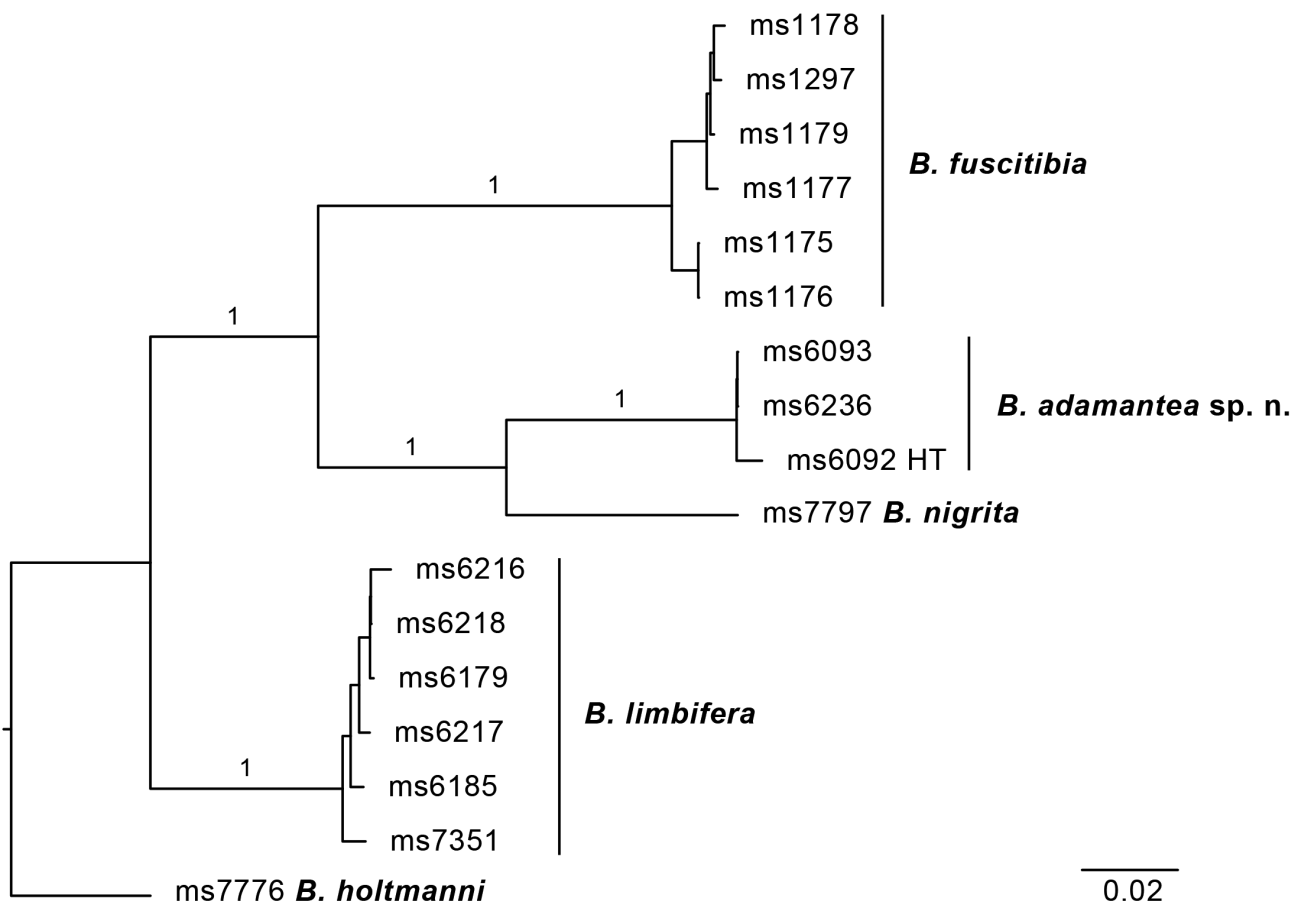
Maximum likelihood tree based on COI sequences of *B.adamantea* sp. n. and several of its genetically closest congeners. Bootstrap branch supports shown for intraspecific relationships. HT = holotype.

*Bactroceraadamantea* is easily distinguished from all other Bactrocera using either section of COI. The closest species is *B.nigrita*, with a minimum intraspecific pairwise distance of 7.82 % in 1,496 bp of COI [8.66 % in COI5P, 7.18 % in COI3P] (Figure 11).

**Figure 12.**
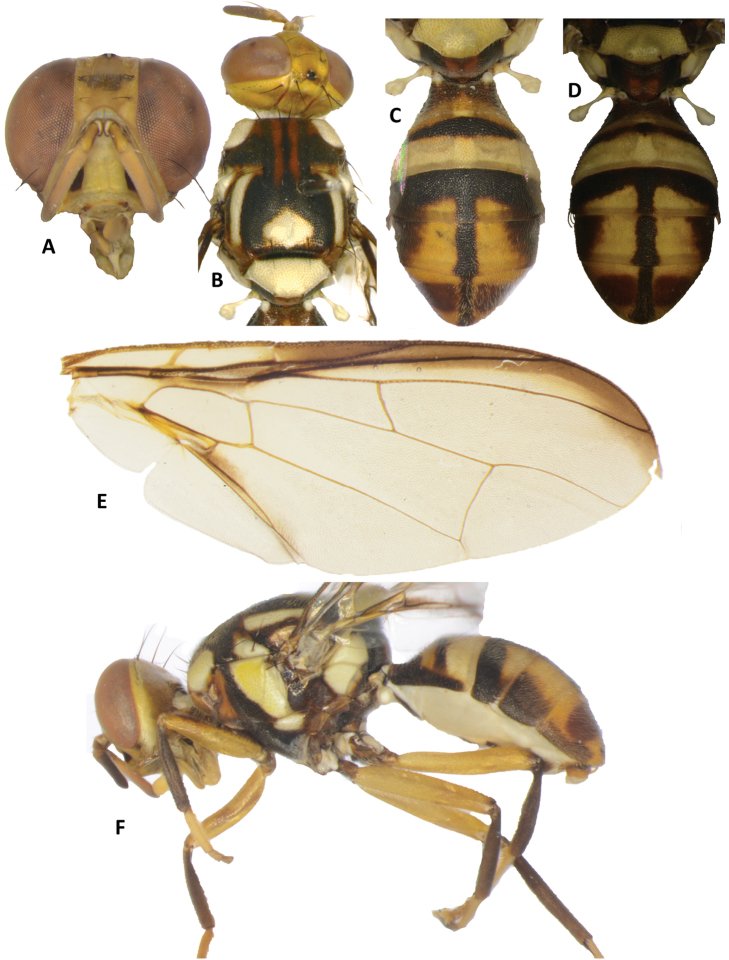
Bactrocera (Bactrocera) adamantea. **A** head **B** head and scutum **C, D** abdomen **E** wing **F** lateral view.

##### Description of adult.

*Head* (Figure 12A). Vertical length 1.66 ± 0.09 (*SE*) (1.55–1.73) mm. Frons, of even width, length 1.38 ± 0.02 (1.36–1.40) times as long as broad; fulvous with light fuscous (may be absent) around orbital setae and on anteromedial hump; latter covered by short red–brown hairs; orbital setae dark fuscous: one pair of superior and two pairs of inferior fronto-orbital setae present; lunule yellow. Ocellar triangle black. Vertex light fuscous. Face fulvous with medium sized oval black spots in each antennal furrow; length 0.58 ± 0.04 (0.55–0.63) mm. Genae fulvous, with fuscous sub-ocular spot; red–brown seta present. Occiput fulvous to light fuscous and yellow along eye margins; occipital row with 6–8 dark setae. Antennae with segments 1 (scape) and 2 (pedicel) fulvous and segment 3 (first flagellomere) fulvous with fuscous outer surface; a strong fulvous dorsal seta on segment 2; arista black (fulvous basally); length of segments: 0.23 ± 0.01 (0.23–0.25) mm; 0.28 ± 0.03 (0.25–0.30) mm; 0.82 ± 0.01 (0.80–0.83) mm.

*Thorax* (Fig. 12B, F). Scutum black except red–brown as two parallel bands running from anterior margin to mid-length and as markings around notopleural suture and medial to postpronotal lobes. Pleural areas black except red–brown proepisternum and anterior portion of anepisternum below postpronotal lobes. Yellow markings as follows: postpronotal lobes; notopleura (notopleural callus); broad mesopleural (anepisternal) stripe, reaching level of anterior notopleural seta dorsally, continuing to katepisternum as a transverse spot, anterior margin slightly convex; anatergite (posterior apex black); anterior 80 % of katatergite (remainder black); two broad parallel-sided lateral postsutural vittae ending at level of intra-alar setae; large diamond-shaped medial marking on posterior end of scutum. Postnotum red–brown medially and black laterally. Scutellum yellow except for narrow black basal band. Setae (number of pairs): 1 scutellar; 1 prescutellar; 1 intra-alar; 1 posterior supra-alar; 1 anterior supra-alar; 1 mesopleural; 2 notopleural; 4 scapular; all setae well developed and red–brown.

*Legs* (Figure 12F). Femora fulvous, except for small fuscous subapical spot on outer surface of fore femur; tibiae dark fuscous; mid-tibiae each with an apical black spur; tarsi fulvous.

*Wings* (Figure 12E). Length 5.78 ± 0.11 (5.67–5.89) mm; basal costal (bc) and costal (c) cells colorless; microtrichia in outer corner of cell c only; remainder of wings colorless except fuscous subcostal cell, moderately broad fuscous costal band overlapping with R_2+3_ and widening slightly as it crosses apex of R_2+3_ to end between extremities of R_4+5_ and M, a narrow fuscous anal streak ending at apex of posterior cubital cell; dense aggregation of microtrichia around A_1_ + CuA_2_; supernumerary lobe of medium development.

*Abdomen* (Figure 12C, D, F). Elongate oval; terga free; pecten present on tergum III; posterior lobe of surstylus short; abdominal sternum V with a deep concavity on posterior margin. Tergum I and sterna I and II wider than long. Tergum I fulvous with dark fuscous along base medially and black along lateral margins; tergum II fulvous except for a narrow transverse black band across anterior margin which extends to cover narrow lateral margins. Terga III–V orange–brown with a dark T-shaped pattern consisting of a broad transverse black band across anterior margin of tergum III expanding broadly over lateral margins and a broad medial longitudinal black band over all three terga, broad black lateral markings on tergum IV and narrowly black along lateral margins of tergum V. A pair of dark fuscous ceromata (shining spots) on tergum V. Abdominal sterna dark fuscous except for fulvous sternite II.

**Etymology.** The name *adamantea* is an adjective that refers to the diamond-shaped marking on the scutum, uniquely distinctive to this species.

**Notes.** It was referred to as *Bactrocera* species 69, sister to B. (Bactrocera) fuscitibia Drew and Hancock, in the phylogeny presented in San Jose et al. (2018). It keys as far as couplet 80, page 240, in Drew and Romig (2016), and differs from *B.lateritaenia* by the presence of the conspicuous diamond-shaped marking on the scutum (Figure 12B), which is also uniquely distinctive among Dacine fruit flies.

## Supplementary Material

XML Treatment for Bactrocera (Tetradacus) ernesti

XML Treatment for Bactrocera (Asiadacus) connecta

XML Treatment for Bactrocera (Parazeugodacus) clarifemur

XML Treatment for Bactrocera (Bactrocera) adamantea

## Supplementary materials

Supplementary material 1 Table S1. Species of Dacine fruit flies recorded in Vietnam and neighboring countries, with references to published records Data type: occurence This is an open access article distributed under the terms of the Creative Commons Attribution License (CC BY 4.0), which permits unrestricted use, distribution, and reproduction in any medium, provided the original author and source are credited.Luc Leblanc, Camiel Doorenweerd, Michael San Jose, Hong Thai Pham, Daniel Rubinoff
